# Study on predictive models for swallowing risk in patients with AECOPD

**DOI:** 10.1186/s12890-024-02908-y

**Published:** 2024-02-23

**Authors:** Shuyun Xiong, You Zhou, Wenfeng He, Jinling Zhu, Wenfang He, Meizhu Ding, Dongxu Si

**Affiliations:** 1 Guangdong Provincial Hospital of Chinese Medicine, 510000 Guangzhou, Guangdong China; 2 Guangzhou Red Cross Hospital, 510000 Guangzhou, Guangdong China

**Keywords:** AECOPD, Dysphagia, Swallowing function, Water swallow test, mMRC

## Abstract

**Background:**

Dysphagia is considered a complication in patients with acute exacerbation of chronic obstructive pulmonary disease (AECOPD). However, AECOPD may have risk factors for dysphagia.

**Methods:**

Through a cross-sectional study, which included 100 patients with AECOPD. General information, Pulmonary function, COPD assessment test (CAT) and modified Medical Research Council (mMRC) were collected by questionnaire. The questionnaires were administered by uniform-trained investigators using standard and neutral language, and swallowing risk was assessed by using a water swallow test (WST) on the day of patient admission.

**Results:**

Among the 100 included patients, 50(50%) were at risk of swallowing. Multivariate analysis using logistic regression analysis showed that age ≥ 74 years old, mMRC ≥ level 2, hospitalization days ≥ 7 days and the use of BIPAP assisted ventilation were important influencing factors for swallowing risk in patients with AECOPD.

**Conclusion:**

Patients with AECOPD are at risk for dysphagia, assessing age, mMRC, hospitalization days and the use of BIPAP assisted ventilation can be used to screen for swallowing risk, thus contributing to the implementation of early prevention measures.

Chronic obstructive pulmonary disease (COPD), a potentially fatal respiratory condition, is defined by persistently restricted airflow. Its occurrence rises significantly with advancing age [1]. The death rate linked to this condition nearly two folded between 1970 and 2000 [2]. By 2030, it was anticipated that this widely occurring ailment would rank as the third most common cause of mortality and be among the top ten contributors to the overall disease burden [3]. 90% of fatalities occurred in nations with lower and moderate income levels [4, 5].

Between 32.7% and 49% of individuals with COPD experience dysphagia, [6–8] and a 33% prevalence of subjective swallowing symptoms in stable COPD [7]. In stable COPD, subjective swallowing symptoms appear to be a prevalent issue. This concern manifests across all phases of the condition, but it is more frequently observed in symptomatic patients and those with reduced physical capacity [7].

Due to the common neuroanatomical mechanisms and pathways involved in respiration and swallowing, precise coordination between them is necessary to protect the airway, which is crucial for safe and effective swallowing function. In addition, both patients with acute exacerbation of chronic obstructive pulmonary disease (AECOPD) and those with heart disease have symptoms of dyspnea. However, individuals in the former encounter notably higher instances of both self-reported and clinically assessed swallowing dysfunction [9].

Patients with AECOPD experience respiratory and swallowing incompatibility, which may lead to more frequent aspiration and deterioration due to the inability to develop airway protection mechanisms [10]. The incidence of aspiration in COPD is 19.9% [11]. In stable COPD, up to 25% of individuals experience aspiration [12], with an inclination towards elevated rates of hospitalizations and mortality over a period of 36 months [13]. The rate of aspiration in patients with AECOPD was 17% [14, 15]. The probability of developing aspiration pneumonia in COPD is 2.4 times higher than that of the general population [16].

The financial impact of dysphagia on inpatient hospitalization is significant, with costs being 40 ∼ 60% higher compared to those without the condition [17]. This has brought a huge economic burden to individuals. Those with dysphagia tend to have prolonged hospital stays, incur higher bills, and are more likely to require post-discharge medical arrangements [18]. This financial strain can have a cascading effect on an individual’s daily life, leading to increased stress, anxiety, and depression, ultimately diminishing their overall quality of life. Moreover, the long-term effects of anxiety and depression are considerable, further exacerbating the decline in quality of life [19, 20]. Within nursing home settings, COPD stands out as the second most influential factor predicting the occurrence of aspiration pneumonia, which is closely linked to dysphagia [21]. Individuals with dysphagia face an elevated risk of mortality compared to those without the condition [18, 22]. Consequently, the interaction between COPD and dysphagia represents a potential contributing factor to the high mortality rates associated with COPD. Early and accurate identification and assessment of swallowing function in patients with AECOPD is critical.

However, the existing research evidence is insufficient to provide valuable evidence for clinicians to identify and evaluate the swallowing function of AECOPD in the early stage [23–25]. This study was designed to screen and evaluate swallowing function in patients with AECOPD by water swallow test (WST) [15], and to screen for relevant swallowing risk factors, and providing reference for clinicians to early evaluate and prevent swallowing risk in AECOPD, as well as develop protective measures, treatment, liquid and nutritional needs.

## Materials and methods

### Participants

Patients with AECOPD hospitalized at Guangdong Provincial Hospital OF Chinese Medicine were included from June 2022 to April 2023. Diagnostic criteria for AECOPD: Based on the patient’s symptoms, signs, chest X-ray or CT, and Pulmonary function examination, in accordance with the 2023 GOLD guidelines [1].

Inclusion criteria: Age ≥ 40 years old; Stable vital signs; Able to eat orally which was screened by functional oral Intake Scale (FOIS) [26]; Normal language communication skills; Able to cooperate in completing assessments and investigations.

Exclusion criteria: Severe cognitive impairment was assessed by mini-mental state examination (MMSE). (Normal: 27–30; mild cognitive impairment: 21–26; moderate cognitive impairment: 10–20; severe cognitive impairment: 0–9) [27]; Patients with dysphagia due to other categorization of disease, such as stroke, central nervous system disease, head and neck tumors, Alzheimer’s disease, Nasopharyngeal carcinoma.

### Ethical approval

Every participant in this study has provided their informed consent, and the research has undergone thorough review by the Medical Ethics Committee of Guangdong Provincial Hospital OF Chinese Medicine.

### Observation and measurement

Observations include gender, age, smoking, underlying diseases, course of disease, use of BIPAP assisted ventilation, Pulmonary function grading (PFG), COPD Assessment Test (CAT) [28, 29], modified Medical Research Council (mMRC) [29–31] and hospitalization days. Predict their association with swallowing risk in AECOPD.

### Assessment of swallowing function

The WST is useful for the early identification of dysphagia and screening for aspiration [32]. In addition, this screening method is simple to operate, harmless and without additional costs [33]. Therefore, in this study, we applied the WST to evaluate and screen for swallowing risk issues in patients [15]. The WST was used for evaluation as shown in Table 1.

### Procedure

The questionnaire survey was conducted face-to-face by attending physicians who received unified training using standard and neutral language, and swallowing function was evaluated with WST on the day of patient admission. Method: Patients were instructed to drink 30 mL of warm water when the sitting position or the head of the bed was raised > 60 º. The researcher observed the process of drinking water for the patient, and recorded whether there was cough, the number of times of drinking water, time consuming.

Grade I: Drinking water once within 5 s without coughing.

Grade II: Drinking water twice or more without choking or coughing.

Grade III: Drinking water at once but with coughing.

Grade IV: Drinking water twice or more times and with coughing.

Grade V: Coughing frequently and cannot drink the water successfully.

Normal: Grade I, less than 5 s; Suspicious: Grade I, more than 5 s or II; Abnormal: grade III ∼ V. According to the WST score, and the included cases were divided into low-risk swallowing group (WST ≤ 1) and high-risk swallowing group (WST ≥ 2).

The questionnaire was filled out anonymously. If the patient is unable to fill it out independently, the investigator will truthfully fill it out on their behalf through question and answer. After completing the questionnaire, it will be retrieved on the spot, invalid questionnaires will be removed, and the questionnaire will be numbered.

### Statistical analysis

Statistical analysis was performed using R software (version 4.1.2, https://www.r-project.org/). Classified variables are expressed as percentages, while continuous variables are expressed as median (interquartile spacing [IQR]). We used Wilcoxon rank sum test, Pearson chi square test, or continuous corrected chi square test to examine inter group differences in baseline characteristics, clinical manifestations, and laboratory data. *P* < 0.05 is considered statistically significant.

Use LASSO regression to quantify the contribution of all potential predictive factors to identify important predictive factors and estimate their impact on swallowing risk without overfitting the data. LASSO regression selects potential risk factors for stepwise regression analysis to determine predictive factors related to final swallowing risk, with a threshold P < 0.05 considered important. Use ‘quality’ for stepwise regression analysis.

## Results

### Characteristics of the research population

**Fig. 1:**
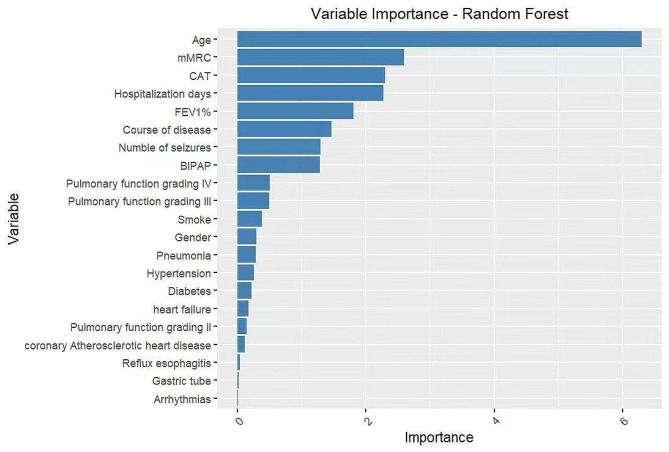
Variable importance sorting. The importance of variables indicates the degree of influence of independent variables on dependent variables. The graph above is a ranking of the importance of variables based on the results of a random forest, arranged from top to bottom. CAT = COPD Assessment Test; mMRC = modified Medical Research Council

Table 2 displays the demographic details of the participants. According to the WST score, the included cases were divided into low-risk swallowing group (WST ≤ 1) of 50 cases and high-risk swallowing group (WST ≥ 2) of 50 cases. Compared with the low-risk group of swallowing, people at risk of swallowing are older (*P* < 0.001), have longer hospital stays (*P* = 0.003), have more acute episodes (*P* = 0.010), have higher mMRC scores (*P* < 0.001), higher CAT scores (*P* = 0.035), are more likely to use BIPAP assisted ventilation (*P* < 0.001), and have poorer Pulmonary function (Grade IV Pulmonary function) (*P* = 0.020). From Fig. 1, it can be seen that the importance calculation results of variables in random forests are age, mMRC, CAT score, disease course, BIPAP, number of acute exacerbations, Pulmonary function, etc.

### LASSO regression analysis

**Fig. 2:**
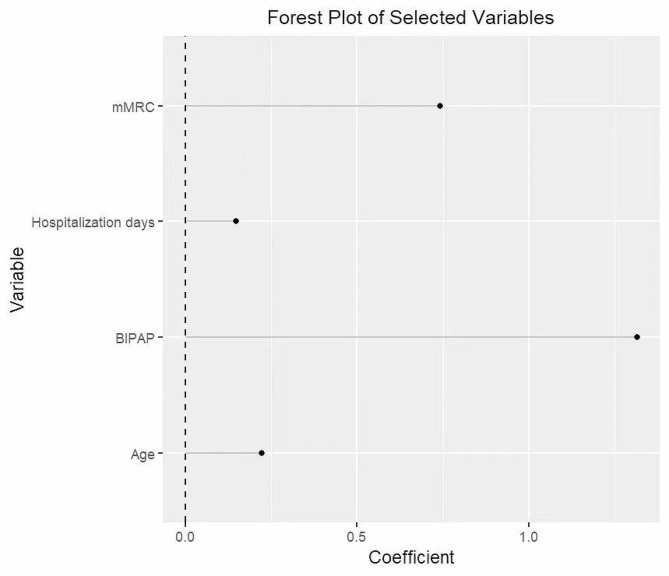
Variable coefficient forest plot. The Forest plot shows the stepwise regression results for swallowing risk, all four predictors increased the probability of swallowing risk. mMRC = modified Medical Research Council

Four potential predictors of swallowing risk were finally identified according to minimum criteria deviation a, minimum absolute contraction and selection operator (LASSO) regression, and stepwise regression, as shown in Table 3; Fig. 2. The regression results showed that the age coefficient was 0.208 and was significant at the level of 1%, which indicated that the probability of swallowing risk increased by 0.208% for each additional year of age, which cutoff value was 74 years, and patients with AECOPD ≥ 74 years had a higher risk of swallowing. The length of hospital stay coefficient was 0.151 and significant at the 5% level, indicating a 0.151% increase in the probability of swallowing risk for each additional day of hospital stay, with a cutoff value 7 days. BIPAP-assisted ventilation coefficient of 1.535 and significant at the 5% level, indicating a 1.535% increased risk of swallowing in patients requiring BIPAP-assisted ventilation. The coefficient for mMRC was 0.790 and significant at the 5% level, indicating a 0.790% increase in the probability of swallowing risk for each 1 grade increase in mMRC. The cutoff value for mMRC was grade 2, and mMRC ≥ 2 had a higher swallowing risk. The Forest plot shows the stepwise regression results for swallowing risk, with all four predictors increasing the incidence of swallowing risk.

### Receiver operating characteristic (ROC) curve analysis

**Fig. 3:**
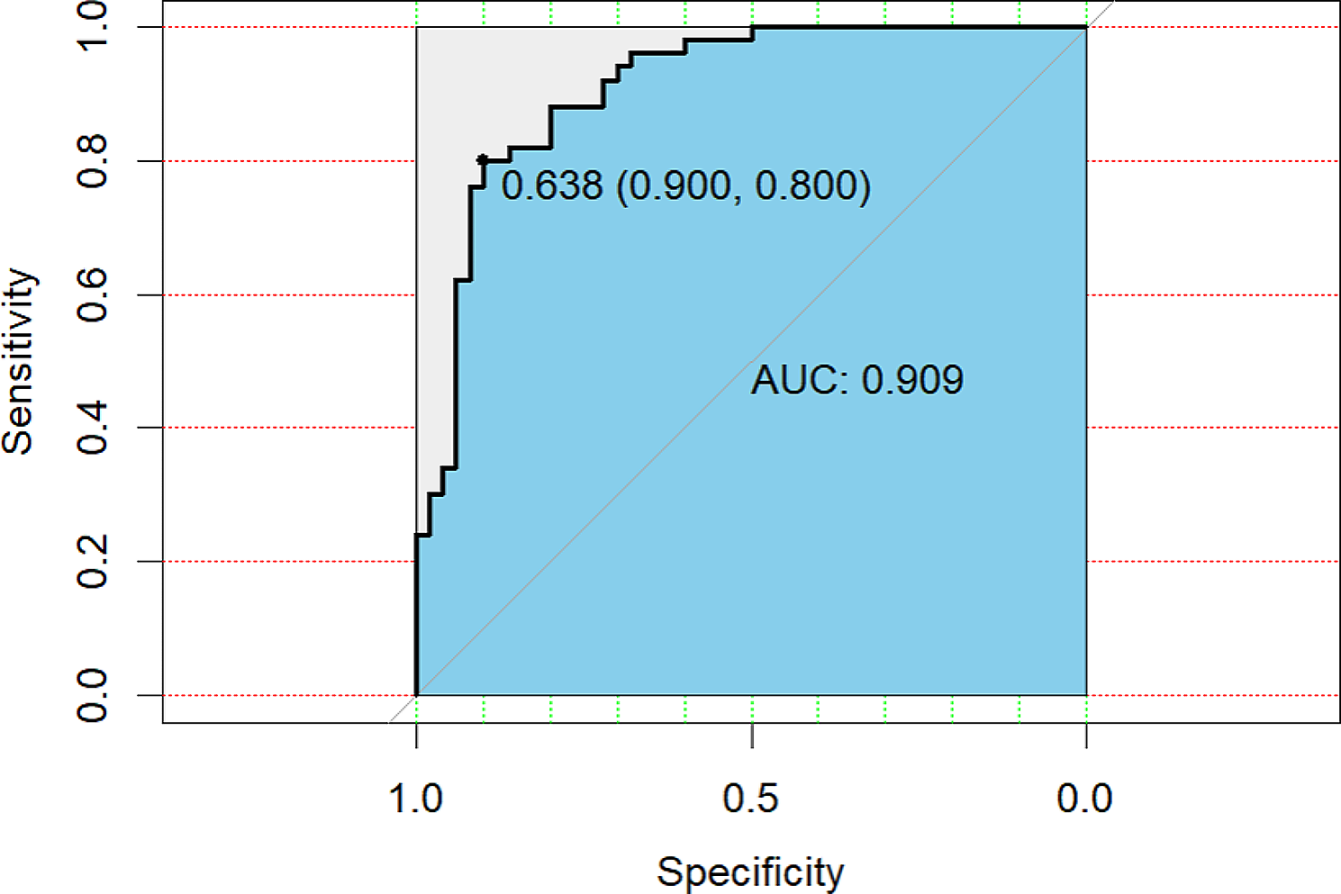
ROC curve of swallowing risk in AECOPD. ROC curves for different models predicting swallowing risk. Clinical parameters included age, length of hospital stay, use of BIPAP assisted ventilation and mMRC. The area under the ROC curve is the prediction performance of the model, The discrimination was low when the AUC was 0.5, moderate when the AUC was 0.6 to 0.8, and good when the AUC was greater than 0.8; AECOPD = acute exacerbation of chronic obstructive pulmonary disease; ROC = Receiver Operating Characteristic; AUC = Area Under Curve; mMRC = modified Medical Research Council

To determine the discriminatory power of the models (i.e., their ability to distinguish patients with and without swallowing risk), we plotted the ROC curve and computed the area under the curve (AUC). AUC equals 0.909, indicating that the model has good performance. Figure 3.

### Calibration of predictive models

**Fig. 4:**
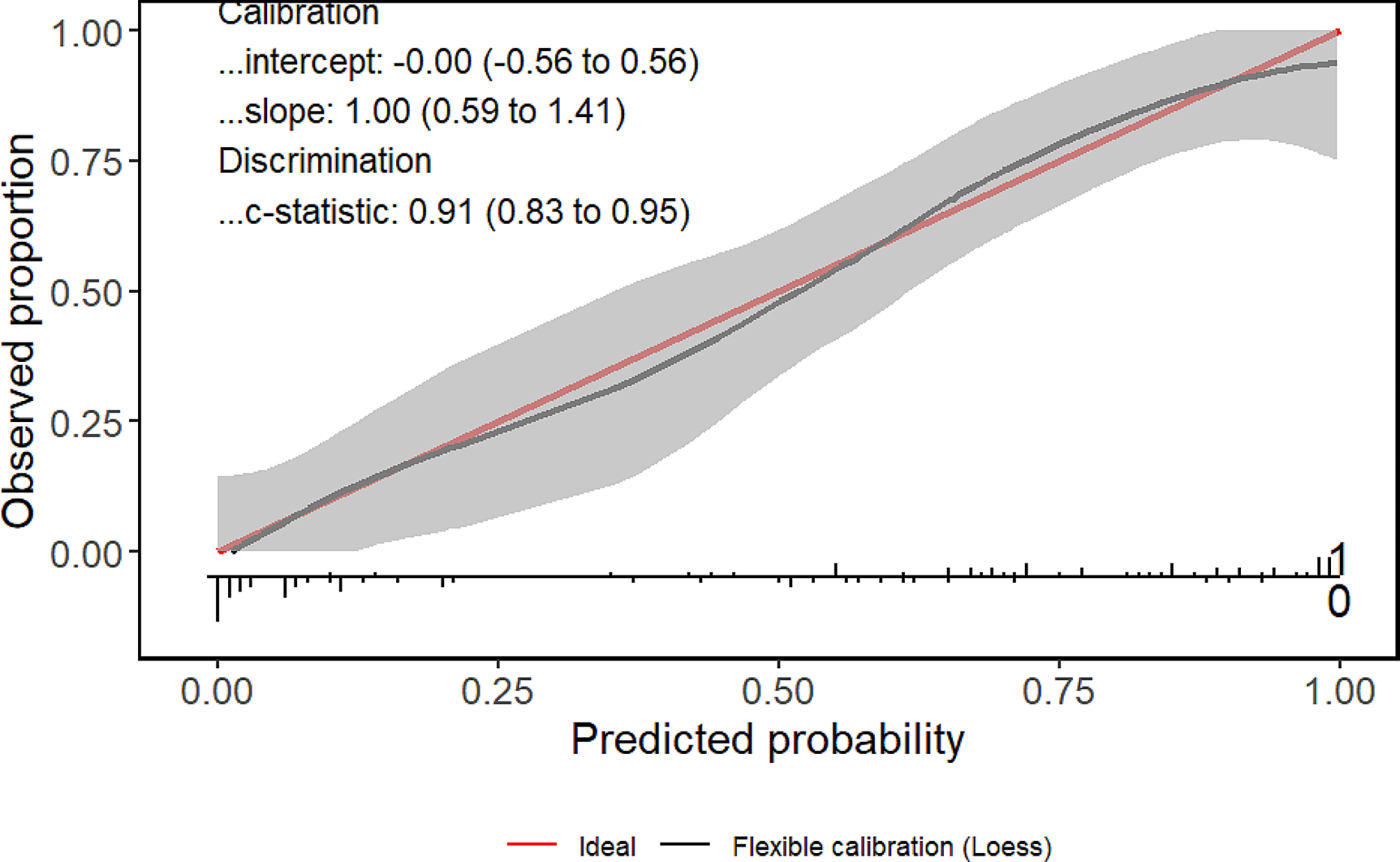
Calibration diagram of prediction model. The horizontal axis of the calibration chart represents the predicted risk of swallowing, while the vertical axis represents the observed actual risk of swallowing, both ranging from 0 to 1. The red line on the diagonal is the reference line, which refers to the situation where the predicted value equals the actual value. The red line is a curve fitting line, and the gray areas on both sides are 95% CI; CI = confidence interval

Subsequently, a calibration chart was used for visual analysis. In this model, the deviation between the calibration curve and the actual curve is very small, indicating strong model performance. Figure 4.

**Table 1:** The WST scale [15]

Grade	Performance
I	Drinking water once within 5 s without coughing
II	Drinking water twice or more without choking or coughing
III	Drinking water at once but with coughing
IV	Drinking water twice or more times and with coughing
V	Coughing frequently and cannot drink the water successfully

WST = water swallow test; Normal: Grade I, less than 5 s; Suspicious: Grade I, more than 5 s or II; Abnormal: grade III ∼ V

**Table 2:** Characteristics of the study population at baseline [n (%)]

Index	WST ≤ 1 *N* = 50	WST ≥ 2 *N* = 50	*p*-value
**Gender**			
Male	40 (80%)	40 (80%)	1.000
Female	10 (20%)	10 (20%)	1.000
**Age**	72.5 (65, 77)	79(76.25, 83)	< 0.001
**Hospitalization days**	6(6, 8)	8(6.25, 12.50)	0.003
**Gastric tube**	3(6%)	0(0%)	0.241
**Course of disease(year)**	8.5 (4, 10)	10 (7.25, 15)	0.066
**Frequency of attacks**	2(1, 2)	2(2, 3)	0.010
**mMRC**	2 (1, 3)	3 (3, 4)	< 0.001
**CAT**	16.5 (11, 22)	21 (16.5, 25)	< 0.001
**FEV1%**	38.4% (32.11%, 52.35%)	35.15% (29.40%, 43.6%)	0.189
**BIPAP**			
Yes	7 (14%)	25 (50%)	< 0.001
No	43 (86%)	25 (50%)	< 0.001
**Smoke**			
Yes	40 (80%)	40 (80%)	1.000
No	10 (20%)	10 (20%)	1.000
**Pneumonia**			
Yes	18(36%)	25 (50%)	0.226
No	32(64%)	25 (50%)	0.226
**PFG**			
I	2 (4%)	3 (6%)	1.000
II	14 (28%)	9 (18%)	0.342
III	23 (46%)	15 (30%)	0.149
IV	11 (22%)	23 (46%)	0.020
**Comorbidity**			
Hypertension	27(54%)	31(62%)	0.543
Diabetes	7(14%)	9(18%)	0.785
Coronary Atherosclerotic heart disease	1(2%)	3(6%)	0.610
Heart failure	3(6%)	9(18%)	0.124
Cardiac Dysrhythmia	0(0)	3(6%)	0.241
Reflux esophagitis	4(8%)	2(4%)	0.674

*P* values for statistical differences between groups according to data characteristics were obtained using the Wilcoxon rank-sum test, Pearson’s chi-square test, or the continuously corrected chi-square test; WST = water swallow test; CAT = COPD Assessment Test; mMRC = modified Medical Research Council; PFG = Pulmonary function grading

**Table 3:** LASSO regression analysis

Index	B	SE	z-value	*P*	OR	95%CI	Cut-off value
Age	0.208	0.051	4.108	< 0.001	1.23	(1.12,1.36)	74
Hospitalization days	0.151	0.074	2.054	0.040	1.16	(1.01,1.34)	7
BIPAP	1.535	0.673	2.282	0.023	4.64	(1.24,17.34)	-
mMRC	0.790	0.317	2.460	0.014	2.18	(1.17,4.06)	2
Constant	-19.636	4.429	-4.434	< 0.001	0.00		

The above table identifies risk factors for swallowing. Four potential predictors of swallowing risk were ultimately identified based on LASSO regression. mMRC = modified Medical Research Council

## Discussion

The act of swallowing is a sophisticated biomechanical process that harmonizes with breathing to safeguard the airway [34]. Yet, in older individuals and those with conditions like COPD, this intricate coordination might not function optimally. When swallowing and breathing are not in sync, it can lead to significant negative outcomes. For instance, individuals experiencing laryngeal penetration are up to four times more prone to developing pneumonia. If pulmonary aspiration takes place, the likelihood of pneumonia increases tenfold [35]. In individuals with COPD, aspiration can happen due to malfunctioning safeguards in the upper airway, decreased harmony between swallowing and breathing, and shifts in breathing patterns brought on by COPD [36]. Although difficulties with swallowing and subsequent aspiration have been acknowledged in COPD for some time, research in this area has been constrained. 56% of COPD patients admitted to the hospital showed positive results in a swallowing screening with WST [9]. Consistent with this, in our study, 50% of patients were at risk of swallowing.

During calm breathing, swallowing tends to occur more frequently during the exhale phase, typically with a moderate-to-low volume of air. This synchronized pattern offers significant biomechanical benefits for both swallowing and safeguarding the airway. It aids in actions such as elevating the larynx, closing the laryngeal vestibule and vocal folds, and opening the cricopharyngeal sphincter [37]. The predominant synchronization between breathing and swallowing involved exhaling-swallowing-exhaling. Grasping the mechanisms that govern this interplay is pivotal in assessing how coordination influences the normal swallowing process in individuals with swallowing difficulties [38].

In normal circumstances when ingesting liquids naturally, there’s a balance struck between the speed and regularity of swallowing, the breathing pattern surrounding swallowing, as well as the rate and volume of respiration. This balance serves the purpose of preventing the risk of pulmonary aspiration [39]. Changes in the coordination of swallowing and breathing could account for the occurrence of pulmonary aspiration and the sensation of breathlessness experienced during swallowing, which is commonly observed in patients with COPD or neurological disorders [40, 41].

Studying the shape changes in rodent swallows through geometric morphometric analysis indicates that the mechanics of swallowing change as animals age. By coupling this with biological tests of age-related adjustments in neuromuscular systems, we can enhance our comprehension of the musculoskeletal issues that underlie swallowing difficulties in the aging process [42].

### AGE

As individuals age, certain oropharyngeal swallowing aspects exhibit distinct alterations. In older individuals, there is a noticeable delay in the onset of swallowing and an extended duration of swallowing apnea, particularly notable with larger boluses. Compared to young counterparts, middle-aged and elderly individuals display a reduced occurrence of expiratory-expiratory respiratory patterns. Additionally, the likelihood of piecemeal deglutition is highest in the elderly and lowest in the young. These findings indicate a gradual shift in the phases of oropharyngeal swallowing as one ages [43]. We found that older patients had higher WST scores, while other factors remained unchanged. Age ≥ 74 years old was the cutoff value for swallowing risk. Age may be a risk factor for swallowing risk in patients. The reason may be that with age, the function and mechanism of swallowing change, such as a decrease in swallowing related muscle strength and weakened tongue pressure, which can lead to the occurrence of swallowing abnormalities. This also suggests that elderly patients may be at high risk of swallowing.

### mMRC

we found a positive correlation between swallowing risk and mMRC in patients, with a cutoff value level 2. The respiratory rate of patients is accelerated and the respiratory cycle is shortened, which makes it difficult to achieve the breath holding time required for normal swallowing, so that swallowing frequently occurs during the transition period from the inspiratory phase to the expiratory phase or during the inspiratory phase, which is prone to poor coordination between breathing and swallowing, and prone to swallowing risk leading to aspiration.

### PFG

Unfortunately, we did not find any further correlation between PFG and swallowing risk. Swallowing problems in stable COPD are related to lower physical abilities, but not to Pulmonary function [7]. A noteworthy inverse relationship exists between AECOPD Pulmonary function and self-reported difficulty in swallowing. However, there isn’t a significant inverse correlation between Pulmonary function and dysphagia identified through clinical screening [9].

### BIPAP

Patients who use BIPAP assisted ventilation have an increased risk of swallowing. It may be related to more severe breathing–swallowing disorders in patients who require BIPAP assisted ventilation. However, Continuous positive airway pressure ventilation (CPAP)can reduce the swallowing risk of AECOPD [44, 45]. Patients with COPD demonstrated segmented swallowing, leading to extended durations for water bolus ingestion. Additionally, they tended to take a breath after each swallow. Compared with spontaneous breathing, swallowing efficiency and the breathing–swallowing pattern improve with CPAP, and dyspnoea decreases during swallowing when using CPAP [37]. In addition, compared with spontaneous breathing and BiPAP, CPAP reduces aspiration risk in patients [44].

### Hospitalization days

Compared with patients with low swallowing risk, patients with high swallowing risk have longer hospitalization days, with a cutoff value of ≥ 7 days. This may be related to the occurrence of aspiration or insufficient nutrient intake.

### Number of episodes and CAT

Finally, in the single factor difference analysis of swallowing risk, the number of episodes and CAT were all related to swallowing risk. However, in the subsequent binary logistic regression analysis, their impact on the risk of swallowing in patients was not statistically significant. This may be because the correlation between variables was not included in the difference analysis. The result of multivariate analysis is the effect of independent and dependent variables after excluding other interfering factors. There is an inseparable correlation between the various factors that affect swallowing risk in patients.

In summary, 50(50%) of the included patients were at risk of swallowing. Age ≥ 74 years old, mMRC ≥ level 2, hospitalization days ≥ 7 days and the use of BIPAP assisted ventilation were important influencing factors for swallowing risk in patients with AECOPD.

Our research has some limitations. To start, it’s important to note that the sample size is limited, potentially introducing selection bias. In the survey, it was found that fewer female patients with AECOPD were included, and this may be related to the limited number of female smokers, which is inconsistent with previous reports; Secondly, instrument evaluation can enhance research.

## Conclusion

Patients with AECOPD have a risk of swallowing, which is related to age, mMRC, hospitalization days, and the use of BIPAP assisted ventilation. Age ≥ 74 years old, mMRC ≥ level 2, hospital stay ≥ 7 days, and use of BIPAP assisted ventilation are high-risk factors for swallowing in AECOPD. Early swallowing risk screening, assessment, and intervention should be conducted to prevent aspiration pneumonia, reduce readmission times, and improve quality of life.

## Data Availability

The datasets used and analysed during the current study available from the corresponding author on reasonable request.

## Abbreviations

AECOPD: Acute exacerbation of chronic obstructive pulmonary disease
CAT: COPD Assessment Test
mMRC: Modified Medical Research Council
WST: Water swallow test
NIV: Noninvasive ventilation
SP: Specificity
PFG: Pulmonary function grading
CI: Confidence interval
ROC: Receiver Operating Characteristic
AUC: Area Under Curve
